# Perioperative Tablet-Based Telemonitoring After Abdominal Wall Hernia Surgery: Pilot Prospective Observational Cohort Study

**DOI:** 10.2196/15672

**Published:** 2020-10-20

**Authors:** Alexander Gräfitsch, Philipp Kirchhoff, Henry Hoffmann, Ralph F Staerkle, Savas D Soysal, Philippe M Glauser

**Affiliations:** 1 Department of General and Visceral Surgery University Hospital Basel Basel Switzerland; 2 Department of Visceral Surgery Kantonsspital Aarau Aarau Switzerland; 3 ZweiChirurgen GmbH-Center for Hernia Surgery and Proctology Basel Switzerland; 4 Visceral Surgery Research Laboratory Department of Surgery Clarunis University Center for Gastrointestinal and Liver Diseases Basel Switzerland; 5 Department of Visceral Surgery Spital Dornach Dornach Switzerland

**Keywords:** tablet-based follow up, day-case surgery, hernia surgery, telehealth, digital health, perioperative activity

## Abstract

**Background:**

Hernia repairs account for millions of general surgical procedures performed each year worldwide, with a notable shift to outpatient settings over the last decades. As technical possibilities such as smartphones, tablets, and different kinds of probes are becoming more and more available, such systems have been evaluated for applications in various clinical settings. However, there have been few studies conducted in the surgical field, especially in general surgery.

**Objective:**

We aimed to assess the feasibility of a tablet-based follow up to monitor activity levels after repair of abdominal wall hernias and to evaluate a possible reduction of adverse events by their earlier recognition.

**Methods:**

Patients scheduled for elective surgical repair of minor abdominal wall hernias (eg, inguinal, umbilical, or trocar hernias) were equipped with a telemonitoring system, including a tablet, pulse oximeter, and actimeter, for a monitoring phase of 7 days before and 30 days after surgery. Descriptive statistical analyses were performed.

**Results:**

We enrolled 16 patients with a mean overall age of 48.75 (SD 16.27) years. Preoperative activity levels were reached on postoperative day 12 with a median of 2242 (IQR 0-4578) steps after plunging on the day of surgery. The median proportion of available activity measurements over the entire study period of 38 days was 69% (IQR 56%-81%). We observed a gradual decrease in the proportion of available data for all parameters during the postoperative course. Six out of ten patients (60%) regained preoperative activity levels within 3 weeks after surgery. Overall, patients rated the usability of the system as relatively easy.

**Conclusions:**

Tablet-based follow up is feasible after surgical repair of minor abdominal wall hernias, with good adherence rates during the first couple of weeks after surgery. Thus, such a system could be a useful tool to supplement or even replace traditional outpatient follow up in selected general surgical patients.

## Introduction

### Background

As health care costs are rising worldwide, member states of the European Union spend on average 10% of their annual gross domestic products on health care [1]. Developing countries are facing even greater challenges due to population growth and lifestyle changes [2]. General surgical procedures are a significant contributor to these expenditures; for example, approximately 20 million inguinal hernia repairs are performed annually worldwide [3].

Due to rising pressure to reduce costs and ongoing efforts to increase patient comfort, more and more surgical procedures have been performed in a day-case setting since the 1990s [4]. The International Association for Ambulatory Surgery encourages that various procedures, including groin hernia repairs, be performed in an outpatient setting [5,6]. Correspondingly, the United Kingdom considers the day-case approach as the standard of care for most surgical procedures [7]. Furthermore, offering outpatient procedures is also encouraged in contemporary guidelines for the repair of groin hernias [8,9]. Switzerland recently started to follow this international trend. However, in 2010, only 8% of all inguinal and femoral hernia repairs were performed as day cases, which is far lower than the rates in France and Sweden with about 62% and 72%, respectively [10].

Although outpatient surgery is considered safe, we question whether surgeons might be losing personal contact with their patients too early [11]. Traditionally, nurses and surgeons have been monitoring complications and encouraging early mobilization in the ward. This inpatient setting will be diminished in the near future in Switzerland, and is already the exception for minor procedures in many countries.

Different approaches utilizing technical innovations such as virtual clinics or electronic devices have been successfully introduced to improve follow up, rehabilitation, and disease management in numerous fields, including for the outpatient management of inflammatory bowel disease, congestive heart failure, or diabetes [12-15]. In cardiovascular surgery, a digital health kit–based follow up after discharge is feasible [16]; however, its use did not reduce the readmission rate after cardiac surgery compared to traditional follow up [17].

### Objectives

We aimed to study the feasibility of tablet-based monitoring perioperative activity 7 days before and 30 days after surgery for minor abdominal wall hernias and to assess whether this could reduce adverse events due to facilitated recognition. Postoperative pain and the occurrence of surgical site infections (SSI) were assessed as secondary outcomes.

## Methods

### Recruitment

Patients undergoing elective open or laparoscopic repair of abdominal wall hernias between October 2017 and September 2018 were eligible for enrollment in this single-center, prospective, observational cohort study. Approval by the regional ethics committee was granted before the study was initiated (Ethics Commission Northwest and Central Switzerland, Project ID 2017-00787) and written informed consent was obtained from all patients. Exclusion criteria were aged below 18 years, emergency procedures, pregnancy or breastfeeding, and inability to use the devices. No remuneration was awarded for participation. The recruitment took place in our outpatient clinic, in which oral and written informed consent was obtained, and the patient was familiarized with the equipment. To gain optimal compliance, the same scientific assistant was responsible for enrollment in all cases and handed out an information leaflet.

### Equipment

A digital health kit (Santigo Telemonitoring Kit, provided by Health In Sight Solutions, GmbH, Munich, Germany) was used in this study. The telemonitoring kit contained a Santiago R tablet, actimeter to be worn on the wrist, and pulse oximeter. The activity was assessed continuously by the actimeter, counting the patient steps per day and per week. The device was equipped with a Swiss SIM card, which provided internet coverage within Switzerland’s national borders.

### Measurement of Parameters

Patients had to measure pulse, blood oxygen saturation, and pain levels at rest, twice daily. The pain level was measured using the visual analog scale (VAS). The actimeter had to be worn continuously. To allow patients to adapt to the measuring routines and to generate a baseline, we set a preoperative observation period of 7 days. As we conducted this study as a pilot trial, we decided to set a postoperative follow-up period of 30 days to gain information about adherence for further trials. As the risk for postoperative SSI in clean procedures is negligible [18,19], we asked participants to send wound pictures only for 7 days after surgery. Furthermore, they were free to take pictures of a suspected wound infection as they wanted. A study assistant monitored the incoming results and data to spot possible complications after surgery. Moreover, this assistant identified adverse events and intervened if requests from the system for data input were ignored.

### Procedures and Follow Up

Procedures were conducted typically with an overnight stay, and the anesthetic regime was left to the discretion of the attending anesthetists. The pain management consisted of nonsteroidal anti-inflammatory drugs. Follow-up appointments in the clinic were not scheduled as we do not see our patients routinely after repair of minor abdominal wall hernias. At the end of the observation period, each patient was asked to fill in a short questionnaire to evaluate the functionality of the provided tablet and actimeter.

### Statistical Analysis

After completion of enrollment, patients’ baseline characteristics such as comorbidities, type of procedure, and length of stay were recorded. Finally, descriptive statistical analyses were performed.

## Results

### Patient Characteristics and Procedures

**Figure 1:**
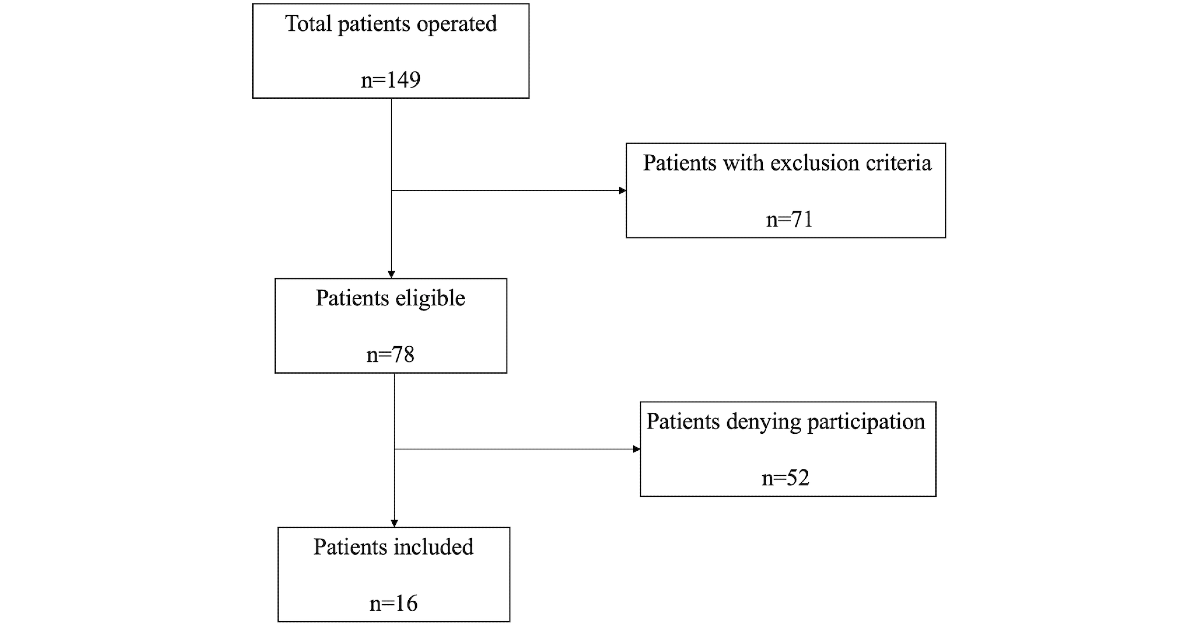
Study flowchart.

We enrolled 16 patients from October 2017 to July 2018, including 11 (69%) men and 5 (31%) women, with a mean overall age of 48.75 (SD 16.27) years (Figure 1, Table 1). Three patients were retired, one patient was currently unemployed, and 12 patients were employees (see Multimedia Appendix 1 for the complete details).

Twelve repairs of groin hernias and four repairs of ventral abdominal wall hernias were performed (Table 2). In cases of trainees delivering the operation, an assisting specialist was always present for supervision.

**Table 1:** Baseline characteristics of the participants (N=16).

Characteristic		Females (n=5)	Males (n=11)
Age (years), mean (SD)		45.80 (11.81)	50.09 (18.31)
BMI (kg/m^2^), mean (SD)		24.80 (2.39)	26.55 (4.93)
ASA^a^ score (1-5), mean (SD)		1.80 (0.45)	2.27 (0.65)
Aspirin^b^, n (%)		0 (0)	1 (9)
**Smoking history, n (%)**			
	Active smokers	3 (60)	4 (36)
	Exsmokers	0 (0)	3 (28)
	Nonsmokers	2 (40)	4 (36)
LOS^c^ (days), mean (SD)		2.40 (0.55)	2.27 (0.47)

^a^ASA: American Society of Anesthesiologists’ classification of physical status.

^b^Ongoing treatment with Aspirin or generic equivalent.

^c^LOS: length of stay in hospital.

**Table 2:** Type of interventions (N=16).

Procedure	Females (n=5), n (%)	Males (n=11), n (%)
TAPP^a^ one side	3 (60)	5 (46)
TAPP both sides	1 (20)	0 (0)
TEP^b^ one side	1 (20)	1 (9)
Lichtenstein repair one side	0 (0)	1 (9)
Direct closure, umbilical	0 (0)	1 (9)
Open sublay repair	0 (0)	1 (9)
Laparoscopic IPOM^c^	0 (0)	2 (18)

^a^TAPP: transabdominal preperitoneal plasty.

^b^TEP: total extraperitoneal plasty.

^c^IPOM: intraperitoneal onlay mesh.

### Activity

**Figure 2:**
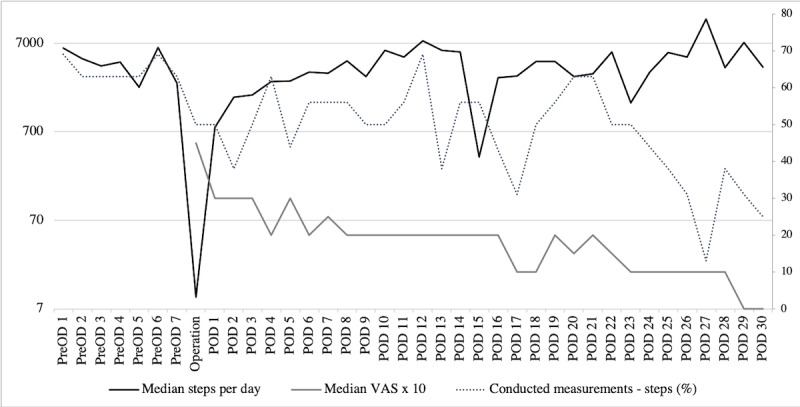
Median steps, median visual analog scale (VAS) for pain, and conducted measurements. PreOD:preoperative day; POD:postoperative day.

Our patients showed a wide range of activity levels over the study period and a considerable amount of activity data were not transferred (Figure 2). Preoperatively, the median step count per day ranged from 2242 (IQR 0-4578) to 6230 (IQR 96-8173) with up to 11 (69%) patients transferring data. Unsurprisingly, daily steps plunged on the day of the procedure but gradually rose from postoperative day 1 and surpassed preoperative levels by postoperative day 12 with a median 7469 (IQR 3314-9126) steps. Subsequently, the step count remained fairly stable, but we noted a remarkable decrease in data transfer over the next few weeks.

With regard to recovering preoperative activity levels, 9 out of the 16 datasets included sufficient information for analysis. Among these patients, 6 (66%) achieved their preoperative levels within 3 weeks after surgery (after 1 week for two patients, after 2 weeks for three patients, and after 3 weeks for one patient).

Pain levels peaked on the day of surgery with a median VAS of 4.5 (IQR 2.25-6) and subsequently decreased over the following weeks with similar rates of transferred data as found for the activity data.

### Pulse Oximeter and SSI

**Figure 3:**
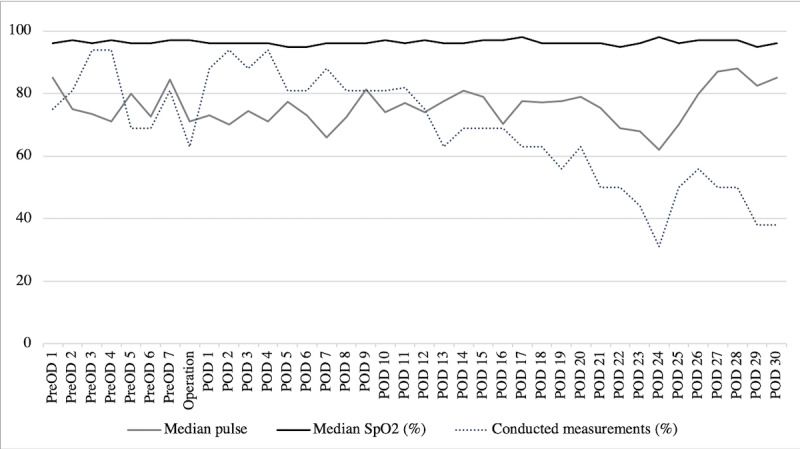
Median pulse, median capillary oxygen saturation (SpO2), and conducted measurements. PreOD: preoperative day; POD: postoperative day.

Average oxygen saturation and pulse levels remained stable throughout the perioperative observation period (Figure 3). Again, the rate of transferred datasets declined steadily, falling below 50% on postoperative day 22. Seven (44%) patients sent wound pictures on postoperative day 3, which was the highest number over the planned 7 postoperative days, but dropped down to as low as 2 (13%) on postoperative day 6. No SSI occurred during the study period.

### General Feedback

Several patients stated that the actimeter was sometimes uncomfortable to wear, and that data transfer from the pulse oximeter and actimeter to the tablet was quite long in some instances. Taking photographs of the wound site was considered to be a laborious task. It was suggested to add a field for further information on pain besides the VAS (eg, pain medication was taken, localization of the pain, quality of pain).

Participants noted varying reasons for missing data input, such as personal and professional commitments abroad, inability to wear the actimeter at work, problems with the photo and VAS apps, and issues with transferring actimeter data. Additionally, one patient received the set on postoperative day 1 and one patient lost his actimeter during the postoperative period.

Nine participants (56%) completed the questionnaire, rating the usability of the tablet interface overall and the different apps as relatively easy (mean 1.8, SD 0.93), rated on a score of 1 (easy) to 5 (difficult). All 9 (100%) patients stated that they would participate in such a trial again and 4 (44%) would recommend friends to take part in studies with this system.

## Discussion

### Principal Results

Our single-center, prospective, observational cohort study showed, in principle, the feasibility of a tablet-based follow up after repair of small hernias of the abdominal wall. The majority of patients achieved their preoperative activity levels within 3 weeks. The usability of the system was rated as relatively easy.

### Limitations

Some limitations of our study have to be considered. The number of included patients was relatively low, and selection bias cannot be excluded. For example, our pragmatic inclusion criterion of “being able to handle a smartphone” has to be mentioned in this context. A setback was that some patients had to travel abroad for professional commitments, while others went on vacations, which led to further loss of data due to the chosen SIM card that was valid only in Switzerland. Additional technical issues such as problems with the connection between the devices or difficulties taking pictures also reduced the transferred data volume. As no SSI occurred, our secondary hypothesis regarding the possible advantages of a photograph-based follow up to minimize the impact of SSI could not be evaluated.

### Comparison With Prior Work

Regarding our primary outcome, we managed to monitor our patients’ activity over the study period. However, surprisingly, the overall completeness of the datasets was quite low; for example, only 25% of the activity datasets were transferred on all 7 days before the procedure. These findings are in sharp contrast to another study in which adherence rates ranged from 59% to 69% for 12 months while monitoring various parameters in patients with chronic conditions [20]. Colleagues studying the use of electronic diaries in patients with chronic pain found even higher rates, with 92% to 96% compliance over a study period of 3 weeks [21]. Interestingly, the rate of the gathered information from the pulse oximeter was consistently higher than that for the actimeter. The reason may be related to technical issues, as the actimeters did not always transfer data to the tablet. Moreover, it had to be worn all the time, in contrast to intermittently using the pulse oximeter. For example, one person was not allowed to wear the actimeter during working hours as a chef.

It is striking that the transferred pulse oximeter information gradually declined as of postoperative day 11 from its highest level of 90% directly after the intervention. In our opinion, this loss of adherence may be due to well-controlled postoperative pain and the return to regular social and professional commitments. These findings are underpinned as 6 out of 9 patients (67%) managed to reach or surpass their preoperative weekly step count within only 2 weeks after surgery. Another possible explanation for the decreasing data transfer may be technical issues reported by the participants.

We found high satisfaction with the system among our patients; additionally, the system’s usability was rated as relatively easy. These findings reflect results of previous studies in which patients showed high acceptance rates for the tested remote monitoring devices [16,17]. As 100% of the patients who filled in the final questionnaire in our trial stated that they would again take part in a trial with this system, we suspect that a shift to an electronic follow up might be feasible on a broad basis.

### Conclusion

Our study shows that a tablet-based follow up with a primary focus on mobilization can be implemented after minor general surgical procedures. Further studies with control groups should be conducted to evaluate possible cost and adverse event reductions compared with traditional follow up. Moreover, we would suggest studying this or similar systems after major abdominal surgery or complications following previous procedures. Finally, smartphones, instead of tablet-based apps, could possibly enhance adherence in younger patients in future trials.

## Appendix

Multimedia Appendix 1 Professions of participants.

## Abbreviations

SSI: surgical site infection
VAS: visual analog scale
