# Supplementary material for: Perioperative Tablet-Based Telemonitoring After Abdominal Wall Hernia Surgery: Pilot Prospective Observational Cohort Study
Source: JMIR Perioper Med. 2020 Oct 20;3(2):e15672. doi: 10.2196/15672 (PMC7709856; doi:10.2196/15672)
Supplement: Multimedia Appendix 1 [file periop_v3i2e15672_app1.docx]

Professions of participants

1. Non-Working
2. Retired
3. Retired
4. Employee (German Railways)
5. Architect
6. Electrician in training
7. Senior Scientist
8. Electrician in training
9. Cook
10. Building technician
11. Engineer
12. Tram driver
13. Employee (non-profit organization)
14. Retired
15. Insurance manager
16. Controller
